# Vaccination in the Light of the Royal British Commission

**Published:** 1898-03

**Authors:** M. R. Leverson

**Affiliations:** Fort Hamilton, L. I., N. Y.


### VACCINATION IN THE LIGHT OF THE ROYAL BRITISH COMMISSION.

M. R. Leverson, M. D., Fort Hamilton, L. I., N. Y.

(Continued from February No., p. 68.)

We return to November 27th, 1889.

Q. 4,512-3 (By Mr. Meadows White). Dr. Cory puts in some tables showing 32,002 cases of calf lymph vaccinations from April, 1882, to end of September, 1889, and of 323 "cases that returned for complaint," viz.: 260 sore arms, 38 eruptions, 16 erysipelas, 8 axillary abscesses, and 1 abscess over the sternum; also 8 cases of death.

Q. 4,527 (By Mr. Picton). The cards before mentioned (Qs. 4,370 and 4,509) are sent out in the cases of vaccination by human as well as by calf lymph.

Q. 4,528. "You told us you found no case of insusceptibility to vaccination; is there such a thing as insusceptibility to small-pox apart from vaccination?" "That would only be an opinion. No; I do not think there is; of course I am supposing the small-pox virus to be used under the same conditions that vaccine is used."

Qs. 4,529-30. He thinks that that is the opinion of medical men generally.

Q. 4,531. Does vaccination create insusceptibility to re-vaccination?" "It does for a certain time afterward."

Q. 4,532. "For how long?" "It varies immensely."

Q. 4,533. "Have you tried to re-vaccinate cases that had been vaccinated?" "Yes."

Q. 4,534. "What is the shortest limit of time within which you find it possible to re-vaccinate after vaccination?" "That depends on what you mean by 're-vaccinating.' Different people have different meanings attaching to the word."

Q. 4,535. "What I mean by re-vaccination is what is ordinarily meant by re-vaccination, the practical appearance of a vesicle after a certain number of days?" "Yes; but are you taking the eighth day? Because many re-vaccinations run the whole course before the eighth day."

Q. 4,537. "What I meant by my question was, What is the longest time after a primary re-vaccination that you have found re-vaccination impossible?" "You are looking at the thing from an entirely different point of view to what I am; any answer I should give you would be a misleading one from your point of view."

Q. 4,538. "I am only asking you for the fact. I only want to know for how long a period of time primary vaccination prevents susceptibility to re-vaccination. You say the time varies?" "It would be a very short time if you looked to results immediately afterward, but a very considerable time if you took the ordinary day for inspection, the eighth day."

Q. 4,539. "I do not quite understand you?" "If you would kindly read this pamphlet I have here, *Some Aspects of the Vaccination Question*, you would perhaps get from it the information you require."*

Q. 4,540 (Sir James Paget). "Would you expect that a person vaccinated last month could be effectually re-vac-

---

*Having attentively studied a copy of this pamphlet, which is a reprint from Volume XV of *St. Thomas' Hospital Reports*, and which I procured from England for the purpose, I confidently assert that it nowhere furnishes an answer to Mr. Picton's very plain question. The only thing approaching an answer is to be found on page 6, as the results of the protracted torture of an infant by eleven vaccinations on eleven successive days; the sum of such results is that all inoculations made during the last four days, *i. e.*, after the eighth day, were "unsuccessful."

It was also found that all the "successful" vaccinations matured on the same day, viz., the ninth. The rest of the pamphlet is a childish pretence at *scientific*, aye, even at geometrical illustration of a *non-sense!*

cinated twelve months hence?" "Yes; a certain result would be produced.

Q. 4,541. Also six months hence.

Q. 4,542. "Three months hence?" "Between three months and six months."

Q. 4,543 (Mr. Picton). "For from three months to six months the human subject would be insusceptible to re-vaccination?" "Yes."

Q. 4,545 (Mr. Picton). "Does small-pox create insusceptibility to small-pox?" "To attacks of small-pox undoubtedly it does, in most people and *for a long time*,* but as regards fatality of that minority of cases that become attacked by small-pox a second time my answer must be no. If you read that paper you will see some facts that throw light upon the point."

Q. 4,546. "I want to have an answer on the minutes. Your answer is no?" "My answer is no; small-pox confers no certain immunity from death by small-pox. A second attack of small-pox when it does occur is sometimes almost as bad as the first."

Q. 4,547. "Does small-pox give insusceptibility for any period of time?" "Yes; for a great many years." (Q. 4,548.) "For more than three months?" "Yes."

Q. 4,550. Inoculation for small-pox and natural small-pox are two separate things, and (Q. 4,551) Dr. Cory regards vaccinia as modified small-pox!

Dr. Cory holds (Q. 4,552) that there is evidence of a difference between natural small-pox and inoculated small-pox; and that after an inoculation of small-pox the system would become as early susceptible to inoculated small-pox as to re-vaccination after vaccination; and, further, that you would have the same kind of complications as you would have with vaccination.

---

*I have been unable to find any evidence to support this view.—M. R. L.

Q. 4,553. Dr. Cory is not speaking from experience; he has never inoculated small-pox into the human subject.*

---

*It is easy to trace in this testimony of Dr. Cory the sort of reasoning which has beguiled him and other followers of Jenner, Koch, and Pasteur into their irrational and unscientific reasoning and methods.

Two ideas dominated the medical profession for centuries, both of them unscientific in character, and without evidence for their support. From the influence of these two ideas even Sydenham and Hahnemann were unable wholly to escape.

The first of these was that by habituating oneself to poisons, first by small and then by gradually increasing doses, the system would become fortified against such poisons; and the other, closely related to it, was that certain diseases are auto-protective. Both ideas influence the old school of medicine to this day, and gave rise to the idea of *syphilizing* people to protect them from syphilis, as well as to vaccination to protect against small-pox (Jenner's cunning trick of calling cow-pox small-pox of the cow causing the idea to prevail that it was so), and have also led to the various nostrums of Koch and Pasteur being received with respect. If antiquity could convert nonsense into sense the first of these superstitions would be most worthy of respect. It dates from the time of Mithridates VI, the great enemy of the Romans, and probably from even earlier times. He being in constant dread of being poisoned, compounded a preparation called "Theriaca," composed of a large number of poisonous drugs, in the hope of thereby rendering himself poison proof. The idea continued not only down to Sydenham's days, but much later; its formula, consisting of fifty ingredients, was to be found in the London pharmacopœia of 1762 under the name of Mithridati," while down to 1866 it was still published in the French Codex under the head of Theriaca Andromachi, consisting of seventy-two ingredients!

The idea of the auto-protection of various diseases still largely prevails, although such evidence as we have tends to the conclusion, viz., that every attack of one disease renders the patient after a short period more liable to a second attack by reason of his lowered vitality, and also more liable to be attacked by any other disease which might be going around. But this idea of auto-protection led to the acceptance of vaccination as a prophylactic against small-pox when people were made to believe that cow-pox was "small-pox of the cow" by Jenner's cunning trick of so calling it, a trick laid bare in all its ignominy by Dr. Creighton in *Jenner and Vaccination*, and by Dr. Crookshank in his *History and Pathology of Vaccination*. Imbued with this idea the supporters of vaccination twist everything to its support, and believe anything, however baseless, which can be made conducive to that end. Hence it is that Dr. Cory believes that Dr. Simpson's lymph was procured by the variolation of a cow on the mere reading of Dr. Murphy's notes (Q. 4,331) of what Dr.

To resume.

Q. 4,569 (By Dr. Collins). Dr. Cory believes he was the author of the report which appeared in the *Eleventh Annual Report of the Local Government Board,* on the earlier operations of the animal vaccination establishment in 1882. (Q. 4,570.) In that he referred to the original source of the Dubreuilh lymph which has since been employed at the animal vaccination establishment.

Q. 4,571. "I suppose I may take it that a thorough investigation as to the nature of that lymph was made before it was introduced?" "*I am quite ignorant of that.* I had the lymph sent to me, and the vaccinations were performed with it."

Q. 4,572. "I understood you to refer to a paper in the *Gazette Hebdomadaire* which contained an account of that lymph?" "Yes." (*Supra,* Q. 4,367 and Q. 4,521.)

Q. 4,573. "Are you familiar with the report in the *Gazette Hebdomadaire?*" "No. I have seen it, but I cannot say that I am familiar with it."

Q. 4,574. "You stated in answer to Q. 4,519 and the two following questions that you were under the impression that so-called spontaneous cow-pox was really due to the inoculation of the cow by a milker suffering from small-pox?" "Yes; it was in answer to a question of Sir James Paget."

Qs. 4,575-76. "You were asked, 'In the case you had your own lymph derived from, you think it probable that some one with small-pox had milked the cow?' and you replied, 'There was small-pox very much about Laforet at the time' (Q. 4,520). Then I asked, 'Is that stated in the *Gazette Hebdoma-*

---

Murphy *supposed* Dr. Simpson had stated! It is needless to point out to any one who has had the smallest training in weighing evidence the worthlessness of any such evidence for any purpose, except as something of a guide to future investigation. But neither to Dr. Cory nor to any one of his assistants or superiors does it seem to have occurred that any investigation was needed, so ready were they to swallow whole any statement which chimed in with their "inherited superstition."

*daire de Medecine et Chirurgie?* (Q. 4,521). A. Yes, I believe so?' I believe so has been added there?" "Yes; I have since added that qualification."

Q. 4,577. "On what grounds did you found the belief that the original source of that so-called spontaneous cow-pox was due to the inoculation from a milker suffering from small-pox?" "I only believed so at the time. It is my general belief that vaccinia is modified small-pox."*

Q. 4,578. "Do you find a specific reference in that number of the *Gazette* to which you refer, stating that small-pox was about at Laforet at the time?" "No; I do not know that I do." (See *supra*, Qs. 4,520-21.)

(In what category of witnesses would an experienced member of the bar class Dr. Cory?)

Q. 4,579. "So that beyond a general belief that cow-pox is referable in all cases to the inoculation of human small-pox, you are not aware of evidence which exists to connect the two in that particular outbreak?" "No; I am not."

Q. 4,586. "Are you aware that the use of the Bordeaux lymph has been discontinued in France?" No; I was not aware of it." (See Q. 4,280.)

Q. 4,587. "Are you aware of suggestions which appeared in print at the time that that outbreak was one of spurious cow-pox?" "No; I am not."

Q. 4,588. "You are not familiar with Dr. Leyet's criticisms upon it?" "No. If it had been a spurious case we should have found it out ourselves."

Q. 4,589. "In what way?" "By the appearance of the vesicle. If you have a cabbage in your garden you know that it is a cabbage, and you do not expect to be poisoned by it."

Q. 4,590 (By Sir James Paget). "Have you had experience

---

*And this is scientific evidence from an eminent man of science—a veritable expert!

of inoculation with what is called spurious cow-pox carried on from calf to calf, or from arm to arm?" "Never."

Q. 4,591. "Have you seen the result of spurious cow-pox by inoculation?" "No; I have not."

Q. 4,592. "You feel that you could detect the difference?" "Yes, certainly, in the human lymph."

Q. 4,593 (Dr. Collins). "Is Mr. Bosquet an authority on vaccination in France?" "Yes."

Q. 4,594. "Are you aware that he states that there is no constant feature in the form, the color, or the areola to indicate the true cow-pox?" "*I am afraid that I am rather ignorant of French literature.*"

Q. 4,595. "Are you familiar with the appearance of true cow-pox in the cow?" "Certainly."

Q. 4,596. "What outbreaks in this country have you investigated?" "We have only had the lymph that we have been using at the station."

Q. 4,597. "I mean what investigations have you made in natural cow-pox in the cow?" "*Not any.*"

Q. 4,598. "Was the Bordeaux lymph ever submitted to what was known as the variolous test?" "No, I think not."

Q. 4,600. "Are you aware that one of the first cases that was inoculated with the Bordeaux lymph in Paris terminated fatally?" "No."

Q. 4,603. "It would not misrepresent your contention to say that you hold that cow-pox never occurs in the cow except as the result of inoculation of the small-pox of man?" "That is my opinion."

Q. 4,604. "I see you say that 'a right understanding of this matter is one of great practical importance, for if it be true that cow-pox is but modified small-pox, then no longer must we regard the human body as a soil foreign to the vaccine virus, but rather the cow's, and thus a weighty argument now used in favor of animal vaccination would have to be transferred to the opposite balance.' Would that represent your present view?" "Yes."

Q. 4,605. Dr. Cory thinks that the difficulties of explaining the facts would be greater if it were held that cow-pox and small-pox were independent diseases.

Q. 4,607. Dr. Ballard takes the opposite view, and attributes the disposition of vaccine lymph to deteriorate to the fact of its being cultivated on a foreign soil.

Qs. 4,608-9. Dr. Cory has had no experience of horse-pox or of equination.

Qs. 4,610-11. As a corollary from Dr. Cory's main contention he believes that if cows were not milked and horses were not shod their respective varioloid diseases (as Dr. Cory terms them) would cease to exist.

Q. 4,613-14. Mares suffer as much as horses from the disease, whereas in cow-pox the females only are infected. Dr. Cory thinks this is because the small-pox is conveyed to the heels of horses while they are being shod, and on the cows' teats while being milked.

Q. 4,615. The vesicles also appear in the mouths of horses, but later than in their heels, which he ascribes to the habit horses have of biting themselves on any part that itches, just as vaccinated children scratch their arms and then their buttocks, producing sores upon the latter.

Q. 4,616. "I suppose you do not happen to be aware that at Bordeaux it was found that the success obtained by inoculation with horse-pox was superior to that which was obtained from the use of the Bordeaux lymph?" "I fancy I remember to have heard something to that effect."

Q. 4,617. Equination is more extensively practiced in France than here.

Q. 4,618. Dr. Cory believes that the rarity of cow-pox is due to the diminution of small-pox.

Q. 4,619. He has glanced through the report on the eruptive diseases of the teats and udders of cows which appeared in a publication by the Agricultural Department of the Privy Council for the year 1888.

Q. 4,620. "Are you aware that as a result of an investigation that was made a large number of cases of cow-pox were discovered in various parts of the country?" "Of what they said was cow-pox, I believe."

Q. 4,621. "Does any evidence exist to show that those cases were not cow-pox?" "Not that I know of."

Q. 4,622. "Was small-pox largely prevalent in this country last year?" "No."

Q. 4,623. "The experiments of the Lyons Commission, conducted by M. Chauveau, were to a large extent contrary to the contention you uphold, were they not?" "They were."

Q. 4,624. Dr. Cory has read the papers of Mr. Fleming, of the War Office, upon the subject.

Q. 4,625. "Is Mr. Fleming an authority upon diseases of cattle and horses?" "He is a veterinary surgeon."

Q. 4,626. "Are you aware that he makes this statement: 'I will now venture to dispute every one of the arguments brought forward to prove that human variola and cow-pox are due to the same virus or are the same disease?' " "Yes."

Q. 4,627. "Are you aware that a communication was addressed in the year 1879 by the Irish Local Government Board to the Galway guardians, intimating that lymph obtained by inoculating the cow with small-pox, if used, would render the operator liable to prosecution?" "I heard of it at the time."

Q. 4,628. Dr. Warlomont is an authority upon animal vaccination.

Q. 4,629. "Are you aware that he contends that it is impossible to convert small-pox into cow-pox by the inoculation of the cow with small-pox?" "I was not aware of it, but, I daresay, it is so."

Qs. 4,648-49. After repeating Q. 4,311, Dr. Collins asks,

"Should I be right in interpreting that answer to mean that the test, and the only test except a microscopical one of the goodness of the lymph, would be practically a retrospective one, having watched the stages that the vesicle had passed through?" "Yes, I think so up to a certain time—the time when the lymph is usually taken. You can then tell very well the character of the vesicle."

Qs. 4,650-51. The microscopic test enables you to be sure that there was no blood with the lymph. If a vesicle bleeds at all the lymph is rejected. The same precaution as to excluding blood is not adopted in the case of animal vaccine.

Q. 4,652. "What is the explanation of the difference of procedure?" "The lymph from the animal is *taken by means of clamps*, which are put on the vesicle of the calf. The calf vesicle is so small that you cannot get the lymph out unless it is squeezed out. This pressure also squeezes out the blood, and you cannot obtain the calf lymph without taking also a certain amount of blood, which is at the same time pressed out of the vesicles by these clamps."

Qs. 4,653-54. Witness does not consider it important to exclude blood in the case of the animal, but that it is advisable in the case of the human subject, because it is contended that syphilis may be conveyed by the blood in the human subject.

Q. 4,656. "What evidence exists to show that syphilis is conveyed by the blood and not by inflammatory lymph?" "I do not know that much exists."*

Q. 4,657. He does not remember any evidence pointing in that direction.

Q. 4,658. "Are you aware that Dr. Ballard states in his essay on vaccination that the perfect character of a vaccine vesicle is no guarantee that it will not furnish both vaccine

---

*There is absolutely none. To suppose there could be is to ignore all that is known of biology, and of the physiology of the blood.—M. R. L.

and syphilitic virus?" "I am not aware of it, but no doubt it is so. I had overlooked that statement."

Q. 4,660. "Would you approve of the statement that the perfect character of the vesicle is no guarantee that it will not furnish both vaccine and syphilitic virus?" "I do not disapprove of it." (See *post*, Q. 8,912.)

Q. 4,661. "He also stated in the year 1868 that 'numerous cases are on record to prove that the vaccine virus and the syphilitic virus may both be introduced at the same spot, and even by the same puncture of the vaccinating lancet, and that in such instances both viruses may take effect, the vaccine vesicle running naturally through its various stages and being succeeded by a chancre on the fall of the crust.' Do you agree with that statement?" "Yes."*

Q. 4,664. "Do I correctly understand you to suggest that there are other cases in which the syphilis is actually communicated by vaccination and the rash makes its appearance in the ordinary sequence?" "There have been a few cases where there has been undoubted conveyance of syphilis by vaccination; but they are very few indeed. In all the authenticated cases there had been a definite interval between the vaccination and formation of chancre, and another interval between the formation of the chancre and the appearance of the secondary symptoms."

Qs. 4,665-8. Dr. Cory is more successful than many other vaccinators, and has had only one failure in primary vaccination in children under ten years of age.

Q. 4,669. Has had very little opportunity of testing whether insusceptibility to primary vaccination is greater in the adult than in the infant.

Q. 4,682. Questioned as to his reason for saying (Q. 4,370), "We hear of almost every bad case," he says: "The

---

*Dr. Cory has supplied a note to this answer, that "he intended only to admit the possibility of such an occurrence." And yet, of this *possibility* Dr. Cory is a living witness; he invaccinated syphilis into himself!

notice to bring back irregular cases is on the card, and as the calf lymph was something new, parents were anxious, of course, to know that their children were not injured in any way."

Q. 4,683. Compliance with the law terminates on the child being brought back for inspection at the eighth day if the results are satisfactory.

Qs. 4,684-5. Had heard of a case vaccinated at Lamb's Conduit Street having been brought before the Commission which had not been brought back.*

Q. 4,686 (Dr. Collins). "In answer to Q. 4,390, speaking of the cases of erysipelas which followed vaccination at the calf lymph station, you stated that there was probably some want

*This case illustrates the worthlessness of the "statistics" of the English Local Government Board, advanced to show how few cases of injury occur among the enormous number of their vaccinations; the following will do the same for New York:

On June 17th, 1897, one of the medical officers of Brooklyn, L. I., attended the school on Fifth Avenue, corner of Ninety-second Street, Brooklyn, to vaccinate the children there. After performing the operation on the children who "presented themselves," he stated he would return in a week to see how his victims were getting along. Up to the evening of June 26th he had not returned.

On that evening I wrote to the Board of Health of Brooklyn to inform them of this fact; also, that while in many of the children operated upon the poison did not "take," that two of them were suffering severely.

In my letter I informed the Board of Health that these two children (whom I named) needed immediate attention, and that I dared not treat them, lest if sinister results should follow it might be ascribed to my treatment.

Absolutely no notice was taken by the Brooklyn Board of Health, and the poor little sufferers were left without any medical attendance. One of them (a boy of 10), after suffering great pain, got better without treatment; the other (a girl of 8) did not. On July 3d I began to treat her and she recovered. As my treatment was homœopathic there is reason to hope that the child is constitutionally cured; but who can say how much her vitality may have been lowered by the poison thrust into her blood!

But the vaccinating officials of New York will swear that in so many million vaccinations they have only heard of two or three cases of sore arms!

of correspondence between them and the Registrar-General's figures; I do not quite understand what you meant?" "That I should wish to strike out."

Q. 4,688. "In those cases in which convulsions caused a fatal termination shortly after vaccination, do you trace any causal connection between the two or not?" "Children often die of convulsions owing to something that irritates the bowels, or to mere indigestion, which is quite sufficient to occasion convulsions, and when a child dies of convulsions after vaccination I do not know that you can attribute the death to the vaccination at all."*

Q. 4,689. "You do not think that among the variety of things that might cause convulsions the inflammatory fever following vaccination is one?" "I think it may, perhaps, now and again have something to do with it, but it would always be doubtful whether the death was not from other causes." †

Qs. 4,690-4. Dr. Cory makes five insertions. There is not much difference in results between five and four, but there is between four and three, and as a place occasionally fails, he wishes to get the full result. He does not agree with Curschmann, who advocates six insertions in each arm, and "certainly not" with the authorities who consider that one mark is as good as a number.

Qs. 4,695-4,703. Has taken the temperature in only four cases—in the rectum; the results were nearly uniform; it rises

---

*In truth children never die *of* convulsions, though they frequently die *in* convulsions. And as Dr. Cory recognizes "mere indigestion" as a cause of fatal convulsions, how much more likely is that to be a cause which the strongest advocates of vaccination admit to be "a real disease" causing a "tumult in the blood" which completely changes the constitution of the patient!

†Since 1881 the vaccinating officials of England and Wales have been forced to admit over fifty deaths annually from vaccination. The real number would be more than ten times fifty.

gradually until on the seventh day; the average is 99.7.* He has not taken the temperature of the calf, but *believes* it to be elevated concurrently with the appearance of the vesicles.

Q. 4,704. He does not think that the treatment will explain all the cases of sore arms, and did not mean to imply that it did by his answer in Q. 4,383.

Q. 4,705. "In answer to Q. 4,491 you state, 'My impression is that you get more sore arms after using calf lymph than after using humanized lymph,' would not that suggest that at any rate some of the sore arms are referable to the lymph rather than to the treatment?" "Yes; partly to the lymph."

Qs. 4,706-8. The cicatrices vary immensely as the result of inoculation with the same lymph in different persons. Some are foveated, some plain, some puckered. Where the inflammation has been great you get a puckered cicatrix; where it has not been so extensive, you get a foveated scar.

Q. 4,709. Thinks that the absence of foveation is an indication that the lymph is not of good quality. He finds a great variation in the cicatrices. Lymph that always produces a plain scar is generally weak lymph.

Qs. 4,711-12. Plain scars result sometimes from vaccination in the witness' practice. He considers the protection in such cases would be less perfect and less lasting, although such cicatrices might have resulted from the use of the best possible lymph.

Q. 4,717. The evidence on which witness founds this opinion is that of the results of re-vaccinating such cases.

Q. 4,718. "In what way does re-vaccination differ when it is performed upon a person with plain cicatrices, and when it is performed upon a person with foveated cicatrices?" "The results are so uncertain that I should be very sorry to

---

*This *scientific* witness has taken no steps to ascertain an easily ascertainable fact, but yet expresses his *belief*, without facts!

8

give a prediction of how the re-vaccination would take, whether in a foveated cicatrix or in a plain cicatrix of previous vaccination; but I think that taking a large number of cases you may say that you generally get less results from re-vaccination in persons with well foveated cicatrices than in persons with plain cicatrices."

